# Spatial differentiation and association between ecosystem service value and landscape ecological risk index in Anning River dry valley

**DOI:** 10.7717/peerj.20914

**Published:** 2026-04-06

**Authors:** Hua Wu, Zelin Zhang, Jianwei Zhou, Xingwang Chen, Yuzhong Kong, Xiangyun Kong

**Affiliations:** 1 School of Ecology and Environment, Xizang University, Lhasa, China; 2 Joint Laboratory of Plateau Surface Remote Sensing, Xizang University, Lhasa, China; 3 School of Engineering, Xizang University, Lhasa, China

**Keywords:** Ecosystem service value, Landscape ecological risk index, Dry valley, Spatialdifferentiation, Anning River Basin

## Abstract

To reveal the changes and future states of ecosystem service value (ESV) and landscape ecological risk index (LERI) in typical dry valley ecosystems in the 21st century, this study utilized high-precision land use data combined with ESV assessment, LERI assessment, bivariate spatial autocorrelation, Geodetector, CA-Markov model, and standard deviation ellipse methods. The spatio - temporal evolution, spatial associations and differentiation characteristics of ESV and LERI in the Anning River Basin from 2000 to 2020 were analyzed, and made predictions for 2030. The results indicated: (1) The ESV in the study area exhibited a decreasing trend from 2000 to 2030, with a cumulative reduction of 1.444 billion CNY, where forest land contributed over 78.46% of the total ESV. The basin presented a spatial pattern of lower ESV in the valley and higher in mountainous areas, dominated by high-value areas (accounting for approximately 41.54% of the total area). (2) LERI showed an overall declining trend during the study period, with a spatial pattern similar to ESV, characterized by low values in valleys and higher values in mountainous regions. The extremely low-risk areas were predominant (approximately 37.00%). (3) The spatial differentiation of ESV and LERI was jointly influenced by natural and economic factors, with agricultural production potential and annual evaporation identified as the dominant factors (*q* values of 0.18 and 0.28, respectively). The center of gravity of ESV distribution moved towards the northeast, whereas LERI shifted to the southeast. (4) ESV and LERI exhibited positive spatial correlations (Moran’s *I* > 0), with high ESV—low LERI as the main LISA cluster (approximately 20.92%). These findings highlight the necessity for adopting tailored ecological protection measures based on the spatiotemporal distribution and evolution characteristics of ESV and LERI to promote sustainable regional ecological development.

## Introduction

Currently, intense human activities driven by rapid economic growth and urbanization have increasingly damaged natural environments and ecosystems (Yi et al., 2018), resulting in severe ecological issues such as air pollution and water scarcity (Xia et al., 2018; Baffoe & Matsuda, 2018), thereby challenging the harmonious coexistence of humans and nature (Lan, Tang & Liang, 2017). Ecosystem service value (ESV) and landscape ecological risk index (LERI), as core indicators assessing interactions between human activities and the natural environment (Xing, Hu & Wang, 2020), are closely related to ecological security assessments (Tian & Gang, 2012). ESV quantifies life-supporting products and services provided directly or indirectly by ecosystems, such as climate regulation, water conservation, and soil retention (Zhou et al., 2024a; Feng et al., 2025). Meanwhile, LERI evaluates potential ecological threats caused by land use changes and landscape fragmentation (Wang, Tan & Fan, 2022), reflecting the regional ecological security status (Xing, Hu & Wang, 2020). Together, they reveal ecosystem health and sustainability, which are essential for guiding ecological conservation policy, resource allocation, and disaster risk prevention (Jia, Tang & Liu, 2020). Human activities significantly affect ESV and LERI, as changes in land use and land cover substantially alter ecosystem structures and functions, leading to degradation in services like environmental purification and hydrological regulation (Arowolo et al., 2018). High-intensity human activities, including urban expansion, agricultural development, and overgrazing, exacerbate LERI, resulting in soil erosion and biodiversity loss (Wang, Tan & Fan, 2022). Such mutual interactions are particularly evident in ecologically fragile dry valley regions like the Anning River Basin, necessitating scientific approaches to elucidate their spatial–temporal dynamics and associations.

Since Costanza et al. (1997) proposed the ESV assessment framework and Xie et al. (2015) refined it by developing a localized equivalent value scale tailored to China’s terrestrial ecosystems, ESV has become a critical metric for evaluating the effectiveness of ecological restoration and sustainable management. This advancement has spurred interdisciplinary research boom in global ecology, environmental science, and economics (Zeng et al., 2024). Current ESV studies primarily adopt two methodologies: physical quantity assessment and value-based assessment (Xie et al., 2017). The latter is widely favored for its advantages of low data dependency, operational simplicity, result comparability, and comprehensive evaluation scope (De Groot et al., 2012; Fu et al., 2022), and has been extensively applied across multiple research scales, including urban agglomerations (Han et al., 2022), ecological reserves (Feng et al., 2025), islands (Fu et al., 2022), and river basins (Zhou et al., 2024a). Furthermore, the integration of CA-Markov and PLUS models has significantly advanced ESV research by enabling predictive analytics for future land-use scenarios (Luan et al., 2024), particularly through their capacity to simulate spatiotemporal dynamics and quantify multi-scenario impacts on ecosystem services. Unlike traditional ecological risk assessments, landscape ecological risk evaluation emphasizes the quantitative characterization of spatial heterogeneity in ecological risks (Fushita et al., 2017), thereby supporting ecosystem management and guiding regional strategies for risk prevention and ecological network optimization. The landscape ecological risk index (LERI) has emerged as a research hotspot in geography and ecology (Wang, Tan & Fan, 2022), with the most widely used method integrating land use dynamics and landscape pattern indices (Fu, 2014). This approach directly reflects ecological risks embedded in landscape composition and structure, evaluates cumulative effects of risk factors, and provides actionable insights for landscape planning to mitigate regional ecological degradation (Peng et al., 2015). Recent studies have transitioned from independent analyses ESV and LERI to exploring their interconnectedness (Jia, Tang & Liu, 2020). The investigation of the relationship between LERI and ESV can effectively link ecosystems with human well-being, systematically mitigate regional landscape ecological risks, enhance regional ecosystem functioning, and thereby provide a scientific basis for formulating ecological security strategies (Wang et al., 2023). These efforts are broadly categorized into two frameworks: a notable advancement is the Ecosystem Service Based Landscape Ecological Risk Assessment (ESRISK) (Cao et al., 2018), which synthesizes terrain, anthropogenic pressures, and ecological resilience to establish a multidimensional evaluation system. This framework positions ESV loss as the central metric for risk quantification. Existing research on ESV-LERI correlations predominantly concentrates on economically developed regions—urban areas (Zhou et al., 2024b), river basins (Liu, Yang & Wang, 2023), and economic belts (Zhang et al., 2023). The results of the study indicate that ESV and LERI have positive or negative spatial correlations (Li & Gao, 2019; Bian, Chen & Zeng, 2022; Zhang et al., 2023). However, significant gaps persist in ecologically sensitive but economically disadvantaged zones (*e.g.*, dry valleys), where analyses of ESV-LERI interactions and their co-evolutionary mechanisms remain limited.

This study focuses on the Anning River dry valley as the study area, aimed at analyzing the spatio-temporal evolution, spatial differentiation, and spatial association characteristics between ESV and LERI under rapid socioeconomic development in dry valleys. The Anning River dry valley is a significant water source in southwest Sichuan Province, integral to the economic growth of the Panxi urban agglomeration. The sustainable development of its ecosystems is vital for economic stability and regional ethnic harmony. Since the 21st century, under the influence of national strategic policies including the Western Development Initiative, Grain for Green Program, urban-rural construction land conversion, and targeted poverty alleviation, the Anning River Basin has witnessed significant economic growth and population expansion (population increased from 1.4001 million to 2.0485 million, while GDP surged from 7.192 billion CNY to 96.051 billion CNY during 2000 to 2020), leading to intensifying human–environment conflicts. Furthermore, intensified land development has exacerbated cultivated land degradation and soil erosion, with natural disasters such as forest fires, mudslides, and landslides occurring with increasing frequency (Tian et al., 2019). The unique valley-mountain terrain gradient, combined with human activities such as agricultural irrigation and mining, further complicates the spatial differentiation mechanisms of ESV and LERI. This study, using multi-model approaches including CA-Markov and standard deviation ellipses based on high-resolution land-use data, investigates the spatiotemporal dynamics, spatial correlation patterns, and driving factors of ESV and LERI from 2000 to 2020 and forecasts conditions up to 2030. This study has addressed the research gap in spatial correlation analysis between ESV and LERI in dry valley through the bivariate spatial autocorrelation model, which not only provides scientific reference for ecological conservation in globally distributed fragile ecosystems with analogous hydrological-climatic conditions but also facilitates the construction of regional ecological security patterns. The novelty of this study lies in the innovative application of the CA-Markov model to forecast the spatiotemporal distribution patterns and spatial interrelationships between ESV andLERI for 2030.

## Material and Methods

### Overview of the study area

**Figure 1:**
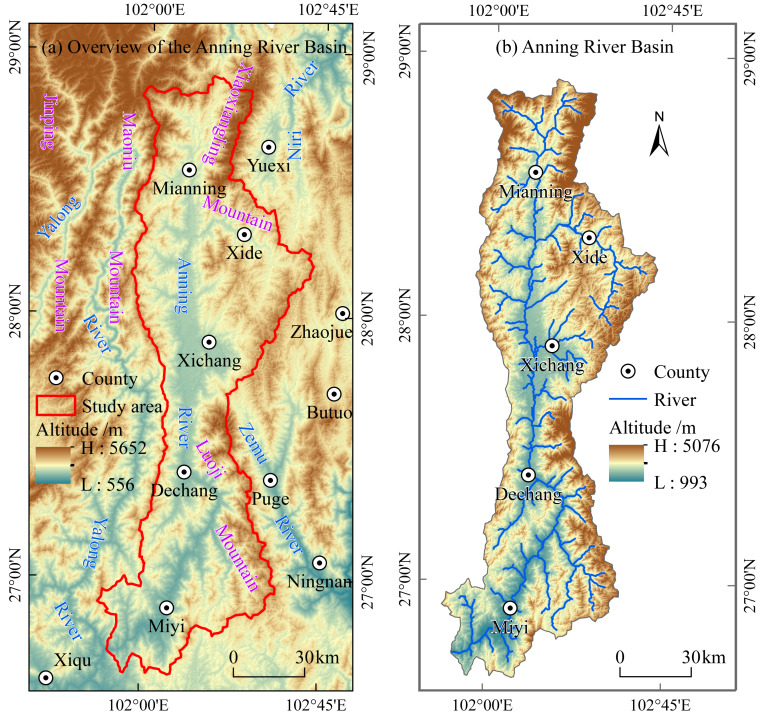
Geographical location map of the Anning River Basin. Note: The base map is produced using the standard map of the Ministry of Natural Resources ( http://bzdt.ch.mnr.gov.cn/browse.html?picId=%224o28b0625501ad13015501ad2bfc0695%22), with the approval number GS (2022) 4314, and no modifications have been made to the map boundaries.

The Anning River originates from Tuowu Mountain of the Xiaoxiangling Range in northern Mianning County, Liangshan Yi Autonomous Prefecture, Sichuan Province, China (Fig. 1). Its the largest tributary on the left bank of the lower Yalong River, a secondary tributary of the Yangtze River, with a total length of approximately 268.20 km. The Anning River Basin covers an area of about 11,053.49 km^2^, extending from 101°47′E to 102°44′E and from 26°36′N to 28°56′N. The Basin is situated in the transitional zone between the Qinghai-Tibet Plateau, Yunnan-Guizhou Plateau, and Sichuan Basin (Shao et al., 2014), exhibiting a topographic gradient declining from the northeast to the southwest with elevations ranging from 993 m to 5,076 m. The Anning River Basin is characterized by a subtropical monsoon climate with abundant solar radiation, an annual average temperature of approximately 12.71 °C, and mean annual precipitation of around 1,133 mm concentrated mainly in summer and autumn (Ma et al., 2023). It represents a typical arid river valley region in southwest China. Additionally, the Basin is an integral part of the Panxi Great Rift Valley within the Hengduan Mountains, serving as a vital ecological barrier in the upper reaches of the Yangtze River (Shao et al., 2014). The alluvial plain along the Anning River, the second largest in Sichuan Province after the Chengdu Plain, features flat terrain, fertile soils, and convenient transportation, making it the political, economic, cultural, and transportation hub of Liangshan Yi Autonomous Prefecture (Luo, Pu & Yu, 2024). Due to significant elevation gradients, complex geological conditions, and its location within a dry valley, the Anning River Basin frequently experiences severe soil erosion and natural hazards such as debris flows and forest fires (Tian et al., 2019).

### Data sources

Data used in this study were acquired as follows: (1) Land use datasets (2000, 2010, and 2020) with a spatial resolution of 30 m were obtained from the GlobeLand 30 platform of the Ministry of Natural Resources of China (https://www.webmap.cn/mapDataAction.do?method=globalLandCover), exhibiting overall accuracy exceeding 83.50% and the Kappa coefficient above 0.78 (Chen et al., 2016), thus meeting the requirements of this study. (2) Annual average temperature, annual precipitation, annual relative humidity, population density, GDP per area, annual mean ground temperature, accumulated temperature ≥10 °C, annual sunshine duration, annual mean wind speed, agricultural production potential, normalized difference vegetation index (NDVI) and annual evaporation data were sourced from the Resource and Environment Science and Data Center, Chinese Academy of Sciences (http://www.resdc.cn/), with a spatial resolution of 1 km. (3) Digital elevation model (DEM) data with 30 m resolution were acquired from the Geospatial Data Cloud platform (http://www.gscloud.cn/), from which slope, aspect, and topographic relief were derived. (4) Data on highways, primary and secondary roads, township administrative centers, rivers, lakes, and reservoirs were obtained from the National Basic Geographic Information Center (https://www.webmap.cn/), using the national basic geographic information database at a scale of 1:1,000,000.

### Research methods

#### CA-Markov model

Cellular Automata (CA) leverage robust spatial computation capabilities to effectively simulate spatial dynamics within complex systems (Zheng & Hu, 2018). The formula is as follows:

(1) \begin{eqnarray*}{C}_{ \left( t+1 \right) }=f \left[ {C}_{ \left( t \right) },N \right] .\end{eqnarray*}

In the formula, *C*represents the finite and discrete set of cell states; *N* defines the neighborhood configuration; *t* and *t* + 1 indicate distinct temporal stages; *f* embodies the local transition rules governing state change.

Markov chain models utilize stochastic process theory to predict future system states by analyzing temporal transition probabilities (Keshtkar & Voigt, 2016). The formula is as follows:

(2) \begin{eqnarray*}{S}_{t+1}={S}_{t}\times {T}_{ij}\end{eqnarray*}

In the formula, *S*_*t*_ and *S*_*t*+1_ represent land use system states at sequential time periods; *T*_*ij*_ denotes the state transition probability matrix.

The CA-Markov model integrates the strengths of CA and Markov chain models (Chen, Sun & Sajjad, 2018), effectively capturing both temporal and spatial dynamics of land use change. In this study, key driving factors—including distance to water areas, distance to roads, distance to township administrative centers, elevation, and slope—were incorporated as spatial constraints to simulate land use transitions. Using the CA-Markov framework, suitability maps were generated to project land use patterns in the Anning River Basin for 2030.

#### ESV Assessment model

Based on the international ESV assessment framework (Costanza et al., 1997) and the equivalent value scale per unit area for terrestrial ecosystems in China (Xie et al., 2017), we revised the ESV coefficients using the average grain yield (5,389.86 kg/ha) of five major counties in the Anning River Basin (Xichang, Xide, Mianning, Dechang, and Miyi) and the 2020 crop procurement price (http://www.lwj.sc.gov.cn/) in Sichuan Province (2.41 CNY/kg). Subsequently, following the theory proposed by Xie et al. (2015)—“The ESV per unit area of farmland equals 1/7 of the market economic value of average grain yield”. Thus, the economic value of the ESV equivalent factor in the study area was calculated as 1,855.65 CNY/ha. Finally, the total ESV was derived using the following formula:

(3) \begin{eqnarray*}ESV=\sum _{i=1}^{m}\sum _{j=1}^{n}{A}_{i}\times {S}_{ij}\end{eqnarray*}

Formula as previously described in Zhou et al. (2025), *ESV* represents the ecosystem service value. *A*_*i*_ represents the area of land category *i* in the evaluation price network. *S*_*ij*_ is the *ESV*equivalent per unit area of *j* ecosystem service types for *i* land types. *i* is the number of land types. *j* represents the type of ecosystem service.

#### LERI assessment model

Based on the landscape structure of regional ecosystems, the comprehensive ecological risk index was constructed by integrating three indices (Landscape disturbance index, landscape vulnerability index, and landscape loss index), in order to analyze the magnitude and spatiotemporal dynamics of ecological risks in the study area (Wang, Yan & Su, 2024). The formula is defined as:

(4) \begin{eqnarray*}LER{I}_{k}=\sum _{i=1}^{n} \frac{{A}_{ki}}{{A}_{k}} {R}_{i}\end{eqnarray*}

In the formula, *LERI*_*k*_ represents the landscape ecological risk index of the *k*assessment unit. *A*_*ki*_ represents area of the *k*land use type within the*i* assessment unit. *A*_*k*_ represents total area of the *k*assessment unit. *n* represents total number of assessment units. *R*_*i*_represents landscape loss index of the *i*land use type. The calculation formulas and definitions of all parameters are provided in Table 1.

**Table 1:** Calculation formulas of landscape indices.

Name	Calculation formula	Description
Landscape separation index *S*_*i*_	${S}_{i}= \frac{A}{2{A}_{i}} \sqrt{ \frac{{n}_{i}}{A} }$	*S*_*i*_ represents the spatial dispersion of patches for landscape type *i*. Higher values indicate greater fragmentation of the landscape. *A*_*i*_ represents total area of landscape type *i*. *A* represents total landscape area.
Landscape dominance index *T*_*i*_	*T*_*i*_ = *mL*_*i*_ + *nP*_*i*_	*T*_*i*_ represents the spatial dominance of landscape type *i*. *L*_*i*_ represents relative density of landscape type *i*. *P*_*i*_ represents relative coverage of landscape type *i*. Weights *m* (0.6) and *n* (0.4) were assigned based on previous study^37^.
Landscape fragmentation index *F*_*i*_	*F*_*i*_ = *n*_*i*_/*A*_*i*_	*F*_*i*_ represents the stability of landscape type *i*. Higher values indicate lower ecosystem stability. *n*_*i*_ represents number of patches in landscape type *i*.
Landscape disturbance index *D*_*i*_	*D*_*i*_ = *aF*_*i*_ + *bS*_*i*_ + *cT*_*i*_	*D*_*i*_ represents the intensity of anthropogenic disturbances on landscape type *i*. Weights *a* (0.5), *b* (0.3), and *c* (0.2) were adopted from prior research^38^. Higher values correlate with reduced landscape stability.
Landscape vulnerability index *V*_*i*_	Obtained by normalization of expert scores^27^.	*V*_*i*_ represents the sensitivity of landscape type *i* to external disturbances. Vulnerability levels (highest to lowest): unused land (6), water bodies (5), cropland (4), grassland (3), woodland (2), built-up land (1). Normalized for analysis.
Landscape loss index *R*_*i*_	*R*_*i*_ = *V*_*i*_ × *D*_*i*_	*R*_*i*_ represents Quantifies ecological losses caused by disturbances to landscape type *i*, comprehensively characterized by integrating landscape disturbance and vulnerability indices.

#### Standard deviation ellipse theory method

The standard deviational ellipse method is a spatial econometric analysis technique that characterizes the spatial distribution and spatiotemporal evolution of geographic elements through key parameters, including center of gravity , azimuth angle, major axis, and minor axis (Feng et al., 2025; Zhou et al., 2025). In this study, the standard deviational ellipse method was employed to analyze the spatiotemporal distribution and evolutionary trends of ESV and LERI in the study area. Key parameters were calculated as follows:

(5) \begin{eqnarray*}\overline{X}& = \frac{\sum _{i=1}^{n}{w}_{i}{x}_{i}}{\sum _{i=1}^{n}{w}_{i}} \mathit{,}\overline{Y}= \frac{\sum _{i=1}^{n}{w}_{i}{y}_{i}}{\sum _{i=1}^{n}{w}_{i}} \end{eqnarray*}

(6) \begin{eqnarray*}\tan \nolimits \alpha & = \frac{ \left( \sum _{i=1}^{n}{w}_{i}^{2}{\overline{x}}_{i}^{2}-\sum _{i=1}^{n}{w}_{i}^{2}{\overline{y}}_{i}^{2} \right) +\sqrt{{ \left( \sum _{i=1}^{n}{w}_{i}^{2}{\overline{x}}_{i}^{2}-\sum _{i=1}^{n}{w}_{i}^{2}{\overline{y}}_{i}^{2} \right) }^{2}+4\sum _{i=1}^{n}{w}_{i}^{2}{\overline{x}}_{i}^{2}{\overline{y}}_{i}^{2}}}{2\sum _{i=1}^{n}{w}_{i}^{2}{\overline{x}}_{i}^{2}{\overline{y}}_{i}^{2}} \end{eqnarray*}

(7) \begin{eqnarray*}{\sigma }_{x}& =\sqrt{ \frac{\sum _{i=1}^{n}{ \left( {w}_{i}{\overline{x}}_{i}\mathit{cos}\alpha -{w}_{i}{\overline{y}}_{i}\mathit{sin}\alpha \right) }^{2}}{\sum _{i=1}^{n}{w}_{i}^{2}} }\mathit{,}{\sigma }_{y}=\sqrt{ \frac{\sum _{i=1}^{n}{ \left( {w}_{i}{\overline{x}}_{i}\mathit{sin}\alpha -{w}_{i}{\overline{y}}_{i}\mathit{cos}\alpha \right) }^{2}}{\sum _{i=1}^{n}{w}_{i}^{2}} }\end{eqnarray*}

(8) \begin{eqnarray*}S& =\pi {\sigma }_{x}{\sigma }_{y}\end{eqnarray*}

Formulas as previously described in Feng et al. (2025), $\overline{X}$ and $\overline{Y}$ represent the coordinates of the centroid. *w*_*i*_ is the weight; *α* is the azimuth of the standard deviational ellipse; ${\overline{x}}_{i}$ and ${\overline{y}}_{i}$ are the deviations of the coordinates of each study object from the mean center; *σ*_*x*_ and *σ*_*y*_ represent the standard deviations along the *x*-axis and *y*-axis, respectively; and *S* denotes the area of the ellipse (km^2^).

#### Geodetector

Geodetector can explore the spatial differentiation characteristics of elements (Wang & Xu, 2017) and use factor detectors and interaction detectors to detect the driving factors and their interactions of ESV and LERI spatial differentiation in the study area. The formula is as follows:

(9) \begin{eqnarray*}q& =1-\sum _{h=1}^{L} \left( \left( {N}_{h}{\sigma }_{h}^{2} \right) \mathit{/} \left( N{\sigma }^{2} \right) \right) =1- \frac{SSW}{SST} \end{eqnarray*}

(10) \begin{eqnarray*}SSW& =\sum _{h=1}^{L}{N}_{h}{\sigma }_{h}^{2}\mathit{,}SST=N{\sigma }^{2}\end{eqnarray*}

In the formula, *q* is the influence of the driving factor on the spatial differentiation of ESV and LERI. *L* is the stratification/partitioning of variable Y or factor X. *N*_*h*_ and *N* represent the number of units in the *h* area and the entire area, respectively. ${\sigma }_{h}^{2}$ and *σ*^2^ are The variance of ESV and LERI for the evaluation grid and study area, respectively. *SSW* and*SST*represent the total variance within the evaluation unit and the total variance across the district, respectively.

#### Bivariate spatial autocorrelation model

The spatial autocorrelation model quantifies spatial interdependencies among geographic elements, comprising global spatial autocorrelation and local spatial autocorrelation (Jia, Tang & Liu, 2020; Liu, Yang & Wang, 2023). To investigate the spatial correlation characteristics of ESV and LERI in the Anning River Basin, this study employed the bivariate spatial analysis module in GeoDa software. The global Moran’s I index was calculated to assess their overall spatial associations and regional disparities. The formula is defined as:

(11) \begin{eqnarray*}{I}_{ESVLERI}= \left[ n\sum _{i=1}^{n}\sum _{j=1}^{n}{{W}_{i}}_{j} \left( \frac{{y}_{iESV}-\overline{{y}_{ESV}}}{{\sigma }_{ESV}} \right) \left( \frac{{y}_{iLERI}-\overline{{y}_{LERI}}}{{\sigma }_{LERI}} \right) \right] / \left( n-1 \right) \sum _{i=1}^{n}\sum _{j=1}^{n}{{W}_{i}}_{j}\end{eqnarray*}

In the formula, *I*_*ESVLERI*_ is the bivariate global spatial autocorrelation coefficient between ESV and LERI. *y*_*iESV*_ and *y*_*iLERI*_ is ESV and LERI values of *i* assessment unit, respectively. *y*_*ESV*_ and *y*_*LERI*_ are the average ESV and LERI for the *i* assessment unit, respectively. *σ*_*v*_ and *σ*_*r*_ are variances of ESV and LERI within the assessment unit, respectively. *n* is total number of assessment units. *W*_*i*__*j*_ is spatial weight matrix based on adjacency relationships. To comprehensively characterize spatial associations among assessment units, local indicators of spatial association (LISA) were employed to visualize localized clustering and dispersion patterns of ESV and LERI (Li & Gao, 2019).

### Results and analysis

#### Spatiotemporal changes in land use

**Figure 2:**
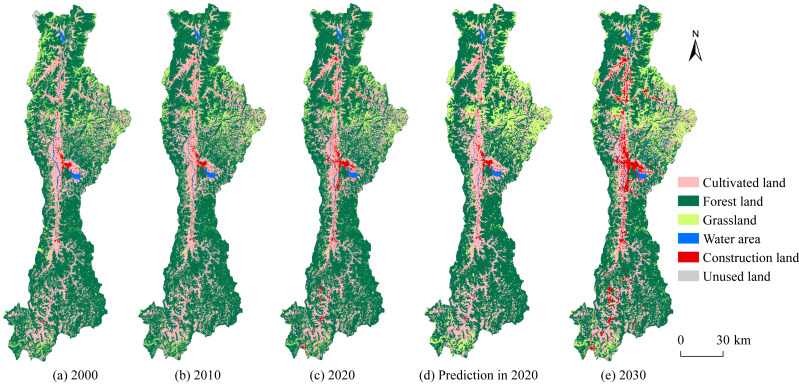
Spatial distribution and changes of land use types in the study area.

To verify the accuracy of the CA-Markov model, the land-use structure of the Anning River Basin in 2020 was predicted using 2010 as the baseline year (Fig. 2). Compared with actual land-use data in 2020, the overall Kappa coefficient reached 0.91, indicating high reliability. Subsequently, using 2020 as the baseline, the land-use pattern in 2030 was projected. From 2000 to 2020, the primary land-use type in the Anning River Basin was forest land (63.02%), followed by cultivated land (25.25%) and grassland (9.11%), while water area, construction land, and unused land each accounted for less than 2.00%. Spatially, land-use types displayed distinct patterns (Fig. 2). Cultivated land and construction land were densely concentrated along flat river valleys of the Anning River and its tributaries. Forest land and grassland predominated on hilly slopes adjacent to the river valleys, whereas water area mainly encompassed Qionghai Lake, Daqiao Reservoir, and major river courses. Unused land was highly concentrated in alpine regions in the northern basin.

**Figure 3:**
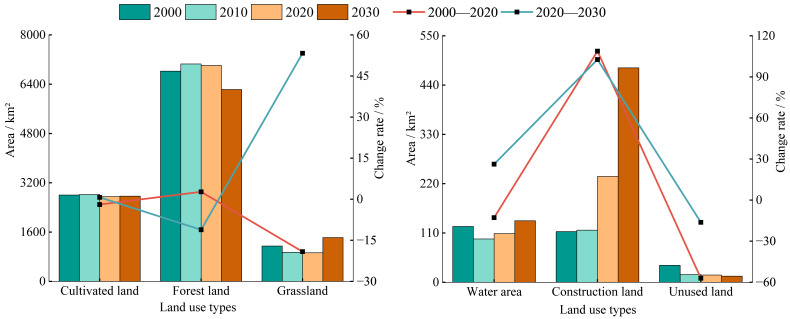
The area changes of land use types in the study area from 2000 to 2030.

Between 2000 and 2020, forest land and construction land increased by 2.70% (184.26 km^2^) and 108.76% (122.99 km^2^), respectively, whereas grassland and cultivated land decreased by 19.13% (219.58 km^2^) and 1.95% (54.62 km^2^), respectively (Fig. 3). For the period 2020–2030, forestland and unused land are projected to decline by 11.15% (781.67 km^2^) and 16.22% (2.63 km^2^), respectively, while grassland, water area, and construction land are expected to increase substantially by 53.30% (494.58 km^2^), 26.26% (28.51 km^2^), and 102.68% (242.39 km^2^), respectively.

#### Changes in ESV

**Figure 4:**
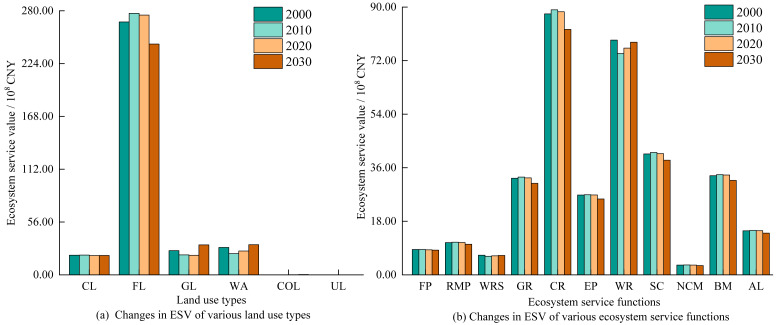
Changes of ESV in the study area from 2000 to 2030. Note: CUL, Cultivated land; FL, Forest land; GL, Grassland; WA, Water area; COL, Construction land; UL, Unused land. FP, Food production, RMP, Raw material production, WRS, Water supply, GR, Gas regulation, CR, Climate regulation, EP, Environmental purification, WR, Water regulation, SC, Soil conservation, NCM, Nutrient cycling maintenance, BM, Biodiversity maintenance, AL, Aesthetic landscapes.

From 2000 to 2020, the total ESV of the Anning River Basin showed a decreasing trend, declining from 34.392 billion CNY in 2000 to 34.218 billion CNY in 2020, and it is projected to further decrease to 32.948 billion CNY by 2030 (Fig. 4). Forest land contributed the most significantly (>78.46%) to regional ESV, followed by water area (8.03%), grassland (7.35%), and cropland (6.10%). Construction and unused lands contributed negligibly (<0.10%). During this period, the ESV of cultivated land, forest land, and unused land decreased, with forest land experiencing a significant decline of 2.348 billion CNY. Conversely, grassland and water area showed increases in ESV by 615 million and 293 million CNY, respectively. Among ecosystem service functions (Fig. 4), climate regulation (25.61%) and hydrological regulation (22.67%) were major contributors, followed by soil conservation (11.86%), biodiversity maintenance (9.74%), and gas regulation (9.48%). All ecosystem service functions decreased from 2000 to 2030, especially climate regulation, gas regulation, and biodiversity, with declines of 522 million, 171 million, and 159 million CNY, respectively.

**Figure 5:**
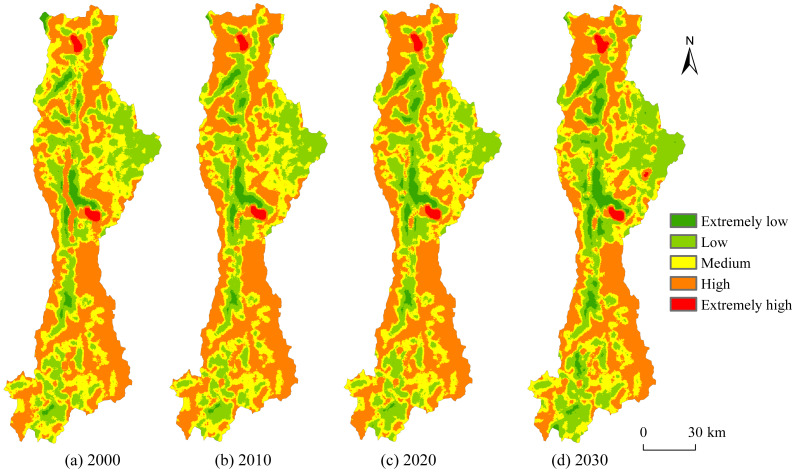
Spatial distribution and dynamics of ESV at different grades.

Calculation of ESV per unit area with 1 km × 1 km evaluation grid study area (10^4^ CNY /ha) to analyze the spatial distribution and temporal dynamics of ESV in the Anning River Basin during 2000–2030. Based on natural breaks classification, the ESV was categorized into five grades: Extremely low [0.15, 1.52), low [1.52, 2.66), medium [2.66, 3.21), high [3.21, 6.57), and extremely high [6.57, 23.11]. Additionally, changes in area proportions for each grades were statistically analyzed. Results (Fig. 5) indicate significant spatial heterogeneity of ESV across the Basin. Extremely low-value areas were highly concentrated along urbanized sections of the main Anning River valley and unused land areas at the northern basin periphery, accounting for approximately 3.22% of the total study area. Low-value areas were mainly located in transitional areas between cultivated land and grassland along tributaries, as well as in rolling hills dominated by grassland along the eastern and southern boundaries, covering approximately 25.29% of the basin. Medium-value areas were primarily distributed within forest-grassland transitional belts in the eastern, southern, and northern parts of the basin, covering about 29.12% of the total area. High-value areas were found extensively within densely forested areas in the northern, southeastern, and western peripheries, accounting for approximately 41.54% of the total area. Extremely high-value areas were distinctly concentrated around major water bodies, such as Daqiao Reservoir, Qionghai Lake, and main tributaries, occupying approximately 0.84% of the total area.

**Figure 6:**
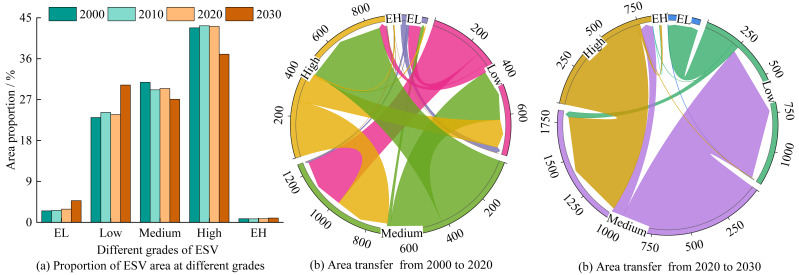
Area proportions and transfers of ESV at different grades/km^2^. Note: EL, Extremely low; EH, Extremely high. The same above.

From 2000 to 2020, the area proportion of medium-value areas decreased by 1.37%, while other value areas exhibited expansion (Fig. 6). Specifically, extremely low-value, low-value, and high-value areas increased by 0.38%, 0.65%, and 0.32%, respectively. The areas converted from medium-valueareas into high-value and low-value areas were approximately 426.83 km^2^ and 260.75 km^2^, respectively. Meanwhile, 265.34 km^2^ and 278.22 km^2^ transitioned from low-value and high-value areas into medium-value areas, respectively. Additionally, substantial area shifts occurred from high-value areas to low-value areas (144.70 km^2^), and from low-value areas to extremely low-value areas (62.51 km^2^). For the period 2020–2030, the areas of medium-value and high-value areas are projected to decline by 2.37% and 6.12%, respectively. Conversely, low-value areas and extremely low-value areas will significantly expand by 6.50% and 1.87%, respectively (Fig. 6). In particular, 964.61 km^2^ of medium-value areas will convert into low-value areas, 742.89 km^2^ of high-value areas will shift to medium-value areas, and 209.10 km^2^ of low-value areas will convert into extremely low-value areas.

#### Changes in LERI

**Figure 7:**
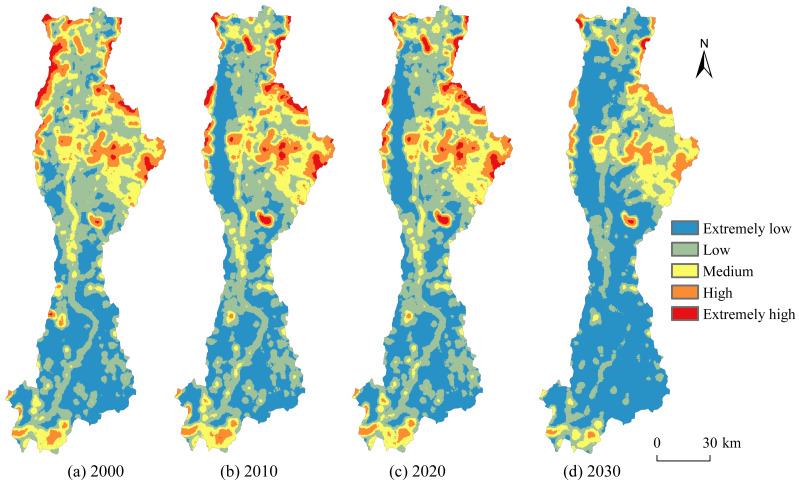
Spatial distribution and dynamics of LERI at different grades.

**Figure 8:**
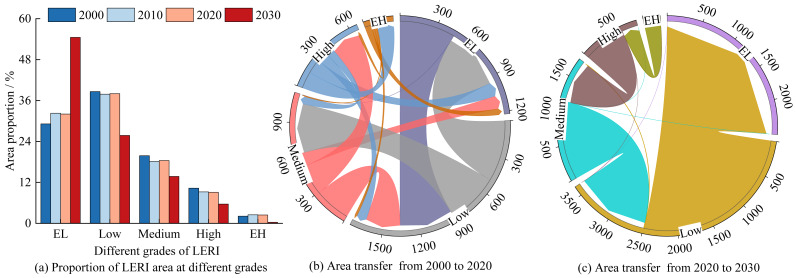
Area proportions and transfers of LERI at different grades/km^2^.

Calculation of LERI with a 1km×1km evaluation grid to analyze its spatial distribution and temporal changes in the Anning River Basin from 2000 to 2030. The LERI values were categorized into five grades using the natural breaks method: Extremely low [0.0043, 0.0141), low [0.0141, 0.0245), medium [0.0245, 0.0404), high [0.0404, 0.0740), and extremely high [0.0740, 0.1604]. The area proportion of each risk grades was then statistically analyzed. The results (Figs. 7 and 8) indicate significant spatial heterogeneity in LERI within the Anning River Basin. Extremely low-risk areas were predominantly concentrated in mountainous forested regions in the western and southern parts of the basin, covering approximately 37.00% of the total basin area from 2000 to 2030. Low-risk areas were mainly distributed in transitional zones between cultivated land and forest land along the Anning River mainstem and its tributaries, accounting for about 35.06% of the total area. Medium-risk areas primarily appeared in the southern and northeastern grassland-dominated areas and in densely populated urban centers along the middle reaches of the Anning River, covering approximately 17.53% of the total area. High-risk areas were concentrated in cropland-grassland transitional areas in the southern and northern basin regions, covering approximately 8.57% of the total area. Extremely high-risk areas were predominantly found in transitional zones between forest land and grassland in the north, as well as around major water area such as Qionghai Lake and Daqiao Reservoir, covering about 1.84% of the total area.

From 2000 to 2020 and further projected into 2020–2030, the area proportion of extremely low-risk areas consistently increased, while the other risk areas exhibited varying degrees of reduction (Fig. 8). Specifically, extremely low-risk areas expanded by 2.90% from 2000 to 2020 and are projected to increase significantly by 22.49% from 2020 to 2030. The area proportions of medium-risk and high-risk areas decreased by 1.42% and 1.22% during 2000–2020, respectively, whereas low-risk and medium-risk areas are predicted to decrease markedly by 12.24% and 4.66%, respectively, during 2020–2030. Between 2000 and 2020, approximately 450.27 km^2^ of extremely low-risk areas and 290.99 km^2^ of medium-risk areas transitioned into low-risk areas; approximately 495.42 km^2^ of low-risk areas and 117.19 km^2^ of high-risk areas transitioned into extremely low-risk areas; around 415.53 km^2^ of low-risk areas and 63.64 km^2^ of high-risk areas converted into medium-risk areas; and 245.14 km^2^ of medium-risk areas shifted to high-risk areas. Between 2020 and 2030, it is anticipated that about 2,484.92 km^2^ of low-risk areas will convert into extremely low-risk areas, 1,142.34 km^2^ of medium-risk areas will shift to low-risk areas, and approximately 617.17 km^2^ of high-risk areas and 238.33 km^2^ of extremely high-risk areas will transition into medium-risk and high-risk areas, respectively.

#### Spatial differentiation of ESV and LERI

**Figure 9:**
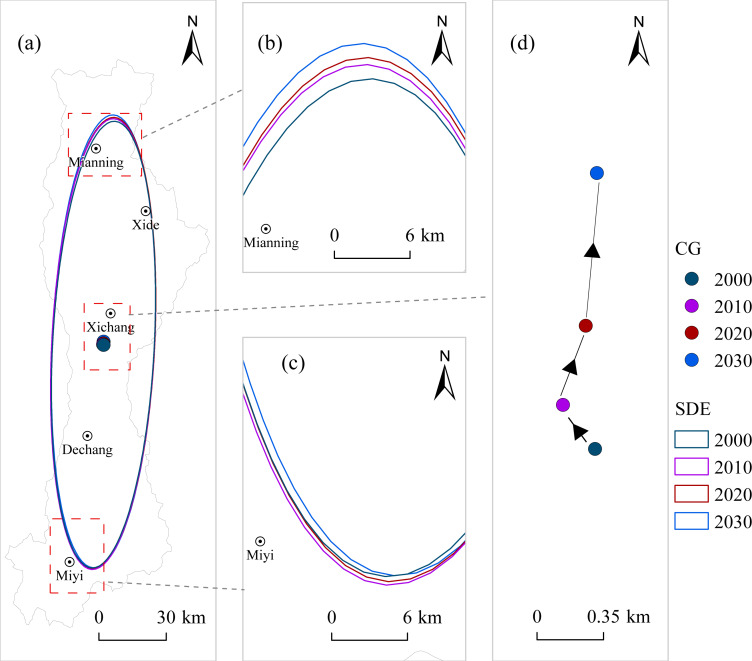
Spatial distribution of standard deviation ellipse and center of gravity of ESV. Note: CG, Center of gravity; SDE, Standard deviational ellipse. The same above.

**Figure 10:**
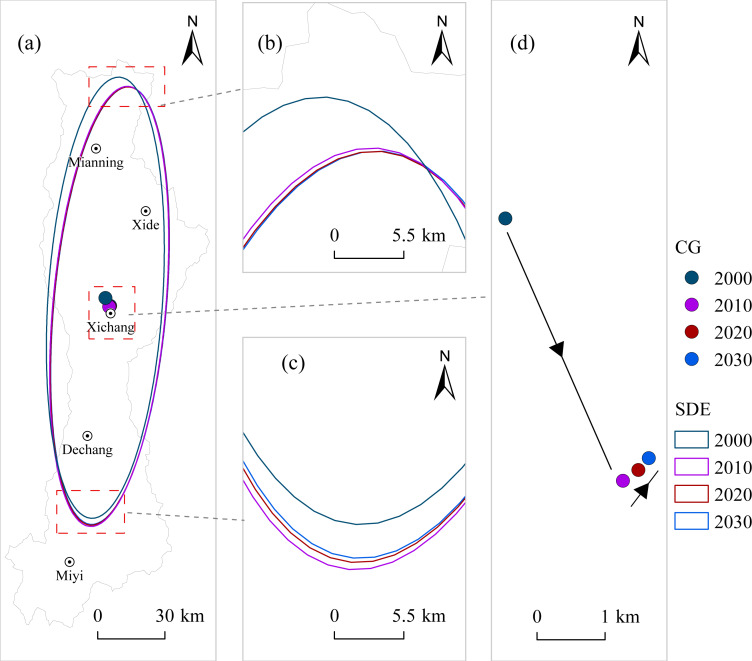
Spatial distribution of standard deviation ellipse and center of gravity of LERI.

Standard deviational ellipse analysis (Table 2, Figs. 9 and 10) indicates that the spatial distribution orientations of ESV and LERI in the Anning River Basin were generally consistent from 2000 to 2030, primarily aligned in a north–south direction corresponding with the Anning River mainstem. This alignment coincides with population and economic development concentration areas, suggesting that ESV and LERI spatial patterns closely correlate with economic and demographic distributions. The major axis length of the ESV ellipse increased from 99.046 km in 2000 to a projected 100.384 km in 2030, while ellipticity fluctuated from 4.338 to 4.366 over the same period (Table 2). This indicates an increasingly significant directional trend in ESV distribution but with decreasing spatial aggregation along the main axis. The minor axis expanded from 22.831 km in 2000 to 23.086 km in 2020 and is expected to slightly decrease to 22.990 km by 2030, suggesting enhanced centralization of ESV distribution during 2000–2020, with a subsequent weakening expected by 2030.

**Table 2:** Changes of standard deviational ellipses and c enter of gravity for ESV and LERI.

Index	Year	Parameters of standard deviation ellipse				Parameters of center of gravity		Distance moved by center of gravity	
		Azimuth angle/^∘^	Major axis/km	Minor axis/km	Oblateness	Longitude (E)/^∘^	Latitude (N)/^∘^	Time	Distance/km^2^
ESV	2000	2.689	99.046	22.831	4.338	102.235	27.769	2000—2010	0.324
	2010	2.516	99.938	23.069	4.332	102.233	27.771	2010–2020	0.450
	2020	2.487	100.075	23.086	4.335	102.234	27.775	2020–2030	0.827
	2030	2.254	100.384	22.990	4.366	102.234	27.782	2000–2030	1.483
LERI	2000	3.438	97.847	25.765	3.798	102.237	27.955	2000–2010	4.230
	2010	4.804	97.781	25.366	3.855	102.256	27.923	2010–2020	0.326
	2020	4.850	97.366	25.265	3.854	102.258	27.924	2020–2030	0.290
	2030	4.861	97.211	25.263	3.848	102.260	27.926	2000–2030	4.150

The centroid of the ESV ellipse generally moved 1.483 km toward the northeast from 2000 to 2030 (Table 2, Fig. 9), with the most notable shift occurring between 2020 and 2030 (0.827 km toward the northeast), indicating improved ecological conditions in the northern region. The major axis of the LERI ellipse decreased from 97.847 km in 2000 to 97.211 km by 2030, with ellipticity increasing from 3.798 to 3.848 during the same period (Table 2). This suggests weakening directional tendencies and increasing spatial aggregation of LERI distribution along the major axis. Meanwhile, the minor axis decreased from 25.765 km in 2000 to 25.263 km by 2030, indicating a gradual weakening of the centripetal trend in LERI distribution. The centroid of the LERI ellipse shifted significantly 4.150 km toward the southeast from 2000 to 2030 (Table 2, Fig. 10), particularly notable from 2000 to 2010 (4.230 km toward the southeast). However, between 2010 and 2030, the centroid is projected to shift slightly toward the northeast, suggesting rising ecological risks in the southeastern part of the basin.

#### Driving factors of the spatial differentiation of ESV and LERI

To explore the driving mechanisms underlying the spatial differentiation of ESV and LERI in the Anning River Basin, a total of 23 natural and socioeconomic variables were selected based on regional environmental characteristics and previous research. These variables include: elevation (X1), slope (X2), aspect (X3), topographic relief (X4), landform type (X5), accumulated temperature ≥10^∘^C (X6), annual evaporation (X7), mean ground temperature (X8), normalized difference vegetation index (NDVI) (X9), annual sunshine duration (X10), annual relative humidity (X11), annual wind speed (X12), annual precipitation (X13), agricultural productivity potential (X14), annual annual temperature (X15), distance to rivers (X16), distance to lakes and reservoirs (X17), population density (X18), GDP per area (X19), distance to primary roads (X20), distance to secondary roads (X21), distance to highways (X22), and distance to township administrative centers (X23).

**Figure 11:**
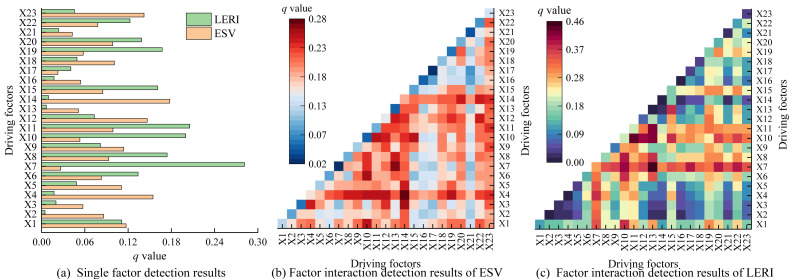
Geodetector results for spatial differentiation driving factors of ESV and LERI. Note: X1, Elevation; X2, Slope; X3, Aspect; X4, Topographic relief, X5, Landform type; X6, Accumulated temperature ≥10° C, X7, Annual evaporation; X8, Annual average ground temperature; X9, NDVI; X10, Annual sunshine duration; X11, Annual relative humidity; X12, Annual average wind speed; X13, Annual precipitation; X14, Agricultural productivity potential; X15, Annual average temperature; X16, Distance to rivers; X17, Distance to lakes/reservoirs; X18, Population density; X19, GDP per area, X20, Distance to primary roads; X21, Distance to secondary roads; X22, Distance to highways; X23, Distance to township administrative centers.

The results of the geographic detector analysis for ESV (Fig. 11A) demonstrated that both natural and socioeconomic factors significantly contribute to the spatial differentiation of ESV. Among them, X14 emerged as the most influential variable, with a *q* value of 0.18. Other key drivers include X4, X12, X23, X1, X9, X5, X18, X11, and X20, each with q values ranging from 0.10 to 0.15. Although the remaining variables have *q* values below 0.10, they still exert varying degrees of influence on ESV distribution. Overall, these results indicated that the spatial pattern of ESV in the study area is jointly shaped by both natural factors (such as topographic relief) and socioeconomic conditions, especially agricultural productivity potential.

Similarly, the spatial differentiation of LERI is also governed by a combination of natural and socioeconomic factors (Fig. 11A). The variable with the highest explanatory power for LERI was X7, with a *q* value of 0.28. Other important contributors include X11, X10, X8, X19, X15, X20, X6, X22, and X1, all with *q* values between 0.11 and 0.21. The rest of the variables have q-values below 0.10 but still contribute to LERI variation to some extent. These findings suggest that the spatial pattern of LERI is influenced by both climate-related variables (*e.g.*, annual evaporation and annual relative humidity) and human factors (*e.g.*, GDP per area and distance to primary roads). Therefore, tailored land-use policies that account for these key factors are crucial to effectively mitigate ecological risks in the basin.

Interaction detection results (Figs. 11B and 11C) further reveal that any two-factor combinations significantly enhance the spatial explanatory power of both ESV and LERI compared to single-factor effects. The interaction between X4 and X14, and between X7 and X13, exerted the strongest joint influence on the spatial distribution of ESV and LERI, with *q* values of 0.28 and 0.46, respectively. Other notable interactions for ESV include X3 with X14, X6 with X10, and X7 with X13, all with *q* values exceeding 0.26. In terms of LERI, strong interactive effects were observed for X7 with X10, X10 with X11, X10 with X12, and X10 with X13, each with *q* values exceeding 0.40. These results underscore that the spatial heterogeneity of ESV and LERI in the Anning River Basin is not solely determined by natural terrain and climatic conditions, but is also strongly influenced by human activity. Consequently, future development strategies in the region should aim to strike a balance between ecological conservation and socioeconomic development. This includes adopting land-use practices tailored to the region’s specific natural and human conditions, and minimizing the adverse effects of unsustainable anthropogenic activities on ecological stability.

#### Spatial association between ESV and LERI

**Figure 12:**
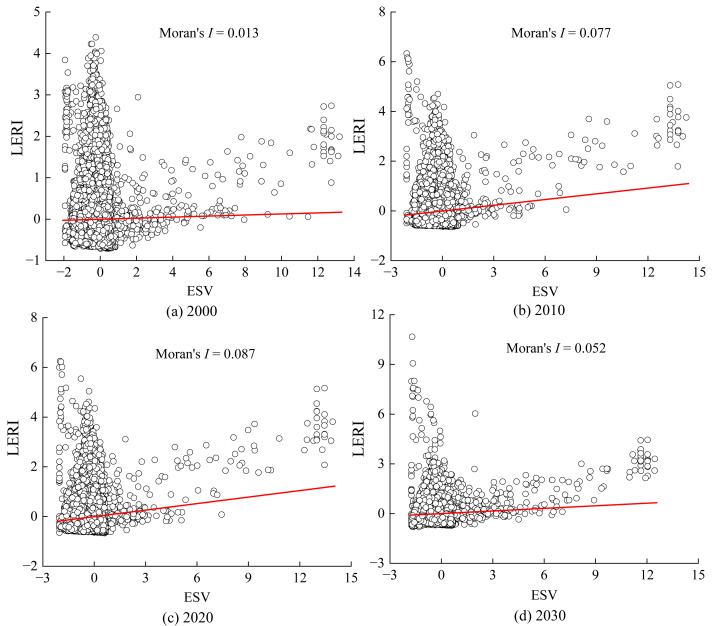
Temporal variations of Moran scatter plots between ESV and LERI.

To explore the spatial autocorrelation between ESV and LERI in the Anning River Basin from 2000 to 2030, both indices were calculated using 1 km × 1 km evaluation units, and their spatial association was analyzed using the GeoDa software. As shown in Fig. 12, the Moran’s I index for the relationship between ESV and LERI increased from 0.013 in 2000 to 0.087 in 2020, and is projected to decline to 0.052 by 2030. These results indicate a positive spatial correlation between ESV and LERI (Moran’s *I* > 0), with the correlation strengthening during 2000–2020, but expected to weaken slightly in 2020–2030.

**Figure 13:**
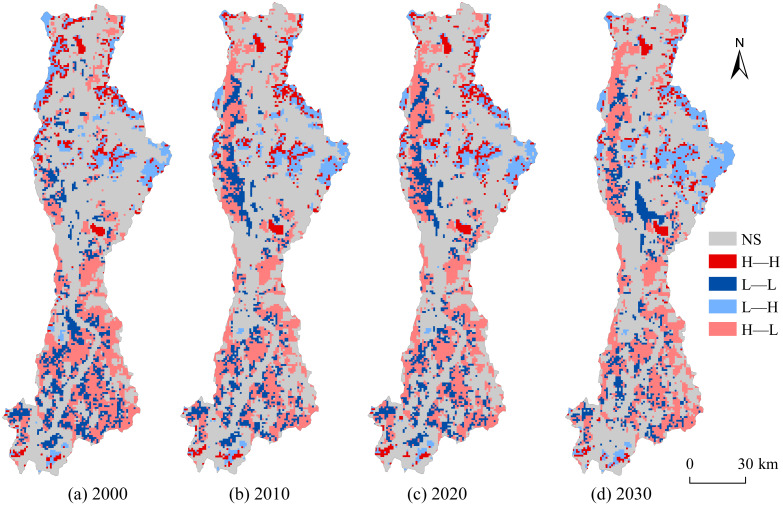
Spatial–temporal patterns of LISA clusters between ESV and LERI.

**Figure 14:**
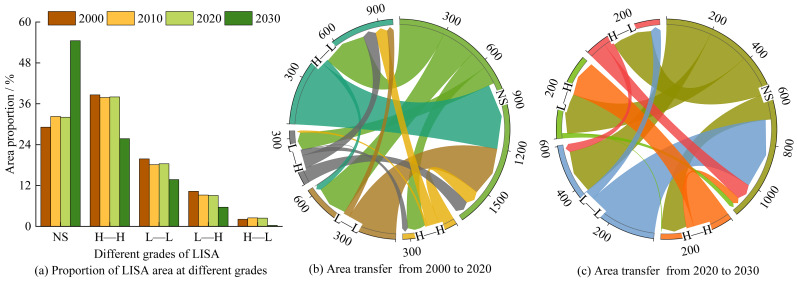
Area proportions and transfers of LISA cluster types/km^2^.

The LISA cluster maps visualize the statistically significant spatial associations between ESV and LERI at the pixel level. The ESV–LERI combinations were categorized into five types: Not significant (NS), High value–High risk (H–H), Low value–Low risk (L–L), Low value–High risk (L–H), and High value–Low risk (H–L). As shown in Figs. 13 and 14, the significant spatial associations are mainly concentrated in the forest and grassland-dominated areas in the southern, western, and northeastern parts of the basin. From 2000 to 2030, the NS clusters accounts for approximately 55.16% of the total basin area, mainly distributed along the mainstem and tributaries of the Anning River and in the northeastern part of the basin. These areas are dominated by cultivated land and construction land, as well as transitional zones between cultivated land and grassland, where ESV and LERI show no significant spatial association. The H–H clusters, occupying 5.03% of the area, is primarily concentrated in the transitional zones between forest and grassland in the northern and southern parts of the basin, and in large water body areas such as Qionghai Lake and Daqiao Reservoir. These areas offer high ecological value but are also highly vulnerable to human disturbances. The L–L clusters, covering about 10.28% of the area, is concentrated in narrow valleys in the northwest and southern parts of the basin, where cropland dominates and both ESV and ecological risk are low. The L–H clusters, occupying 8.61% of the area, is concentrated in the northern part of the basin and represents transitional zones with low ecological value but high ecological risk due to frequent human interference. The H–L clusters accounts for 20.92% of the area, mainly located in mountainous regions in the west and southeast, where extensive forested areas provide high ecological value and relatively stable ecosystems, thus indicating lower ecological risk.

From 2000 to 2020, the areas of NS clusters and H–L clusters expanded by 2.05% and 0.09%, respectively, while other cluster types contracted. Specifically, L–L clusters and L-H clusters both decreased by 0.89%, and H–H clusters shrank by 0.36% (Fig. 14). During this period, frequent transitions occurred between NS clusters and other types: 120.08 km^2^ of NS clusters were converted to H–H clusters, 225.65 km^2^ to L–L clusters, 116.74 km^2^ to L–H clusters, and 293.78 km^2^ to H–L clusters. Conversely, 89.19 km^2^ of H–H clusters, 329.47 km^2^ of L–L clusters, 91.75 km^2^ of L–H clusters, and 446.98 km^2^ of H–L clusters were reverted to NS clusters. Notably, substantial conversions from H–H (80.97 km^2^) and L–H clusters (86.97 km^2^) to H–L clusters were also observed. For the 2020–2030 period, the areas of L–H and H–L clusters are projected to expand by 3.01% and 1.10%, respectively, while NS, H–H, and L–L clusters are expected to contract by 2.17%, 0.75%, and 1.19%. Projections indicate that 116.05 km^2^ of NS clusters will transition to H–H clusters, 200.00 km^2^ to L–L clusters, 184.55 km^2^ to L–H clusters, and 183.55 km^2^ to H–L clusters. Additionally, significant reversions are anticipated: 33.91 km^2^ of H–H clusters, 323.23 km^2^ of L–L clusters, and 76.25 km^2^ of H–L clusters will revert to NS clusters. A notable conversion from L–L to H–L clusters (44.17 km^2^) is also forecasted.

## Discussion

### Unique spatial association between ESV and LERI in dry valley

This study reveals that the spatial association between ESV and LERI in the Anning River dry valley differs from those observed in other arid regions. While most studies in arid areas report a significant negative correlation between ESV and LERI (Jin et al., 2021; Lu et al., 2023), our findings demonstrate a predominantly positive spatial correlation in the Anning River Basin (Moran’s *I* > 0). This divergence may be attributed to the distinct topographic gradient and land-use configuration of dry valleys (Zhang et al., 2014). Within the ecotone created by the “valley–mountain” gradient in the study area, high-ESV areas—such as forest-concentrated regions in the north and south—are generally associated with low LERI values, likely due to limited human disturbance and greater ecological stability in mountainous ecosystems (Cao et al., 2018) . However, localized negative associations persist in the northeastern agro-pastoral ecotone, where cropland expansion coincides with increased ecological risk. This study demonstrates that the development planning of the Anning River Basin must adopt a tailored territorial spatial planning approach that accounts for regional variations in natural conditions and socio-economic characteristics (Wen et al., 2024), thereby effectively mitigating regional ecological risks and promoting harmonious coexistence between humanity and nature. The coexistence of “high value–low risk” and “low value–high risk” patterns reflects the inherent complexity of ecological management in arid valleys. Unlike humid regions such as urban agglomerations or lake basins (Jia, Tang & Liu, 2020; Zeng et al., 2024), ESV–LERI interactions in dry valleys are more susceptible to topographic barriers and non-linear effects of local anthropogenic activities, highlighting the need for spatially adaptive ecological governance strategies (Hu et al., 2019).

### Unique spatial differentiation between ESV and LERI in dry valley

The spatial differentiation of ESV and LERI is influenced by a combination of natural and anthropogenic factors (Yang et al., 2022). In this study, natural factors—including agricultural productivity potential, topographic relief, annual evaporation, and annual relative humidity—remained the predominant determinants of ESV and LERI dynamics, primarily through their modulating effects on landscape configuration and ecosystem processes that drive value- and risk-related spatial patterns (Straton, 2006). Furthermore, interactive effects among these factors exhibited stronger explanatory power for spatial heterogeneity compared to isolated single-factor analyses (Cheng et al., 2022). Spatial analysis revealed a pronounced altitudinal zonation pattern, characterized by elevated ESV/LERI values in mountainous terrain and depressed values in valley systems. Ecological barriers in northern, southeastern, and western forest/grassland zones and their buffer areas demonstrated high susceptibility to anthropogenic disturbances, manifesting as severe fragmentation and serving as primary ecological risk sources. To address these vulnerabilities, future management should prioritize consolidating afforestation outcomes while imposing strict grazing controls in grassland areas, enhancing tourism regulations, and improving reservoir ecological governance. Comprehensive understanding of these cascading ecological consequences is imperative for formulating resilient ecosystem management strategies (Kang, Jia & Zhang, 2022). Regional development must align with ecosystem carrying capacities, ensuring that service utilization remains within sustainable functional thresholds to preserve ecological integrity (Peng et al., 2017).

### Influence of human activities and policy on ESV and LERI

Changes in ESV and LERI in the Anning River Basin are primarily driven by the dual forces of accelerated urbanization, population growth, and ecological policy implementation (Luo, Pu & Yu, 2024). Between 2000 and 2020, the expansion of construction land (an increase of 108.76%) and encroachment upon water bodies (a decrease of 12.81%) directly led to a decline of 1.444 billion CNY in ESV. Meanwhile, reforestation policies such as “Grain for Green” (2000–2020) contributed to forest area expansion, which partially mitigated ESV losses (with a 0.01 billion CNY increase from 2010–2020). The spatial manifestation of policy intervention *versus* anthropogenic pressure is evident: urban sprawl and infrastructure projects—such as the Chengdu–Kunming railway—have exacerbated landscape fragmentation and raised LERI. Conversely, the “One Core, Two Axes, Three Belts, Four Zones” development plan, by delineating ecological redlines (Zhou et al., 2024c), is expected to enlarge the area of extremely low-risk zones by 22.49% during 2020–2030. Furthermore, the Sichuan Province Territorial Spatial Plan for the Anning River Basin (2022–2035) emphasizes an “ecological-first, green development” strategy, which may curb the future southeastward expansion of high-risk areas by restricting intensive development. Nonetheless, major projects such as the Leshan–Xichang Highway could introduce disturbance during construction and post-operation tourism pressure, potentially threatening high-ESV mountain ecosystems (Geneletti & Dawa, 2009). Balancing ecological priorities and developmental demands thus requires a robust dynamic monitoring mechanism to ensure coordinated ecological protection and economic advancement (Wang et al., 2021). For example, governments may mitigate localized ecological risks by implementing green space expansion measures in urban areas and along major road construction projects (Guo et al., 2024), which are characterized by high-human-activity zones.

### Limitations and future outlook

Using high-resolution land use data, this study integrated models including ESV and LERI evaluation, bivariate spatial autocorrelation, Geodetector, CA-Markov, and standard deviation ellipse to analyze spatiotemporal evolution, spatial association, and spatial heterogeneity of ESV and LERI in the Anning River Basin from 2000 to 2030. However, some limitations remain. First, the ESV evaluation relied on the equivalent factor method developed by Xie et al. (2015), which, while widely used in China, does not fully account for the unique ecological service functions of high-altitude ecosystems in the Anning River Basin. Future studies should incorporate field-based observations to calibrate region-specific parameters and improve the accuracy of ESV assessments. Second, the LERI model emphasizes landscape metrics (*e.g.*, patch separation, fragmentation), but lacks representation of dynamic ecological risks such as geological hazards (*e.g.*, debris flows) (Tian et al., 2019) and biological invasions (*e.g.*, *Eupatorium adenophorum*) (Chen et al., 2021). Future work should adopt tools like the InVEST model or ecological network analysis (Guo et al., 2025; Dade et al., 2025) to comprehensively assess the drivers of LERI. Additionally, both ESV and LERI evaluations rely partly on expert judgment, which introduces subjectivity (Xie et al., 2015; Cui et al., 2018). Meanwhile, the predictive accuracy of the CA-Markov model for land-use simulation was exclusively validated through Kappa coefficient analysis in this study. Thus, future research should systematically address systematic prediction errors and epistemic uncertainties by incorporating Bayesian calibration frameworks and machine learning integration, while expanding multi-scenario simulations that incorporate socio-economic policy variables (Chen et al., 2025). The use of 1 km × 1 km resolution data limits the ability to detect fine-scale spatial heterogeneity in steep slopes and riparian zones. Integrating UAV remote sensing and in-situ sensor data (Alvarez-Vanhard, Corpetti & Houet, 2021) can enhance spatial resolution of the data.

## Conclusion

(1) The ESV in the study area exhibited a decreasing trend from 2000 to 2030, with a cumulative reduction of 1.444 billion CNY, where forest land contributed over 78.46% of the total ESV. The basin presented a spatial pattern of lower ESV in the valley and higher in mountainous areas, dominated by high-value areas (accounting for approximately 41.54% of the total area). (2) LERI showed an overall declining trend during the study period, with a spatial pattern similar to ESV, characterized by low values in valleys and higher values in mountainous regions. The extremely low-risk areas were predominant (approximately 37.00%). (3) The spatial differentiation of ESV and LERI was jointly influenced by natural and economic factors, with agricultural production potential and annual evaporation identified as the dominant factors (*q* values of 0.18 and 0.28, respectively). The center of gravity of ESV distribution moved towards the northeast, whereas LERI shifted to the southeast. (4) ESV and LERI exhibited positive spatial correlations (Moran’s *I* > 0), with high ESV—low LERI as the main LISA cluster (approximately 20.92%).

## Supplemental Information

10.7717/peerj.20914/supp-1 Supplemental Information 1 Raw data

## Additional Information and Declarations

### Data Availability

The following information was supplied regarding data availability: The raw data is available in the Supplemental File. The data is available at OSF and Zenodo: - https://osf.io/8s2fd/files/5c36p
- Zhou, J. (2026). Supplemental Files—raw data [Data set]. Zenodo. https://doi.org/10.5281/zenodo.18814446

## References

[ref-1] Alvarez-Vanhard E, Corpetti T, Houet T (2021). UAV & satellite synergies for optical remote sensing applications: a literature review. Science of Remote Sensing.

[ref-2] Arowolo AO, Deng X, Olatunji OA, Obayelu AE (2018). Assessing changes in the value of ecosystem services in response to land-use/land-cover dynamics in Nigeria. Science of the Total Environment.

[ref-3] Baffoe G, Matsuda H (2018). A perception based estimation of the ecological impacts of livelihood activities: the case of rural Ghana. Ecological Indicators.

[ref-4] Bian J, Chen W, Zeng J (2022). Ecosystem services, landscape pattern, and landscape ecological risk zoning in China. Environmental Science and Pollution Research.

[ref-5] Cao Q, Zhang X, Ma H, Wu J (2018). Review of landscape ecological risk and an assessment framework based on ecological services: ESRISK. Acta Geographica Sinic.

[ref-6] Chen F, Chen J, Wu H, Hou D, Zhang W, Zhang J, Zhou X, Chen L (2016). A landscape shape index-based sampling approach for land cover accuracy assessment. Science China Earth Sciences.

[ref-7] Chen L, Sun Y, Sajjad S (2018). Monitoring and predicting land use and land cover changes using remote sensing and GIS techniques—a case study of a hilly area, Jiangle, China. PLOS ONE.

[ref-8] Chen X, Wang G, Peng P, Li J, Shi S, Zhang T (2021). Effects of taxonomic and phylogenetic diversity of resident Pinus yunnanensis communities on Ageratina adenophora invasion in the Panxi region, Sichuan Province. Biodiversity Science.

[ref-9] Chen G, Zhang D, Zhao J, Zhang L (2025). Land use and climate change-based multi-scenario simulation of ecosystem service trade-offs/synergies: a case study of the central Yunnan urban agglomeration, China. PLOS ONE.

[ref-10] Cheng W, Shen B, Xin X, Gu Q, Guo T (2022). Spatiotemporal variations of grassland ecosystem service value and its influencing factors in Inner Mongolia, China. Agronomy.

[ref-11] Costanza R, d’Arge R, De Groot R, Farber S, Grasso M, Hannon B, Limburg K, Naeem S, O’Neill RV, Paruelo J, Raskin RG, Sutton P, Van Den Belt M (1997). The value of the world’s ecosystem services and natural capital. Nature.

[ref-12] Cui L, Zhao Y, Liu J, Han L, Ao Y, Yin S (2018). Landscape ecological risk assessment in Qinling Mountain. Geological Journal.

[ref-13] Dade MC, Bonn A, Eigenbrod F, Felipe-Lucia MR, Fisher B, Goldstein B, Holland RA, Hopping KA, Lavorel S, Le Polain De Waroux Y, MacDonald GK, Mandle L, Metzger JP, Pascual U, Rieb JT, Vallet A, Wells GJ, Ziter CD, Bennett EM, Robinson BE (2025). Landscapes—a lens for assessing sustainability. Landscape Ecology.

[ref-14] De Groot R, Brander L, Van Der Ploeg S, Costanza R, Bernard F, Braat L, Christie M, Crossman N, Ghermandi A, Hein L, Hussain S, Kumar P, McVittie A, Portela R, Rodriguez LC, Ten Brink P, Van Beukering P (2012). Global estimates of the value of ecosystems and their services in monetary units. Ecosystem Services.

[ref-15] Feng B, Zhou J, Hu L, Liu Z, Yang Y, Yang S, Ni J, Bai W, Zhao S (2025). Land use and ecosystem service value spatiotemporal dynamics, topographic gradient effect and their driving factors in typical alpine ecosystems of the East Qinghai-Tibet Plateau: implications for conservation and development. Ecology and Evolution.

[ref-16] Fu B (2014). The integrated studies of geography: coupling of patterns and processes. Acta Geographica Sinica.

[ref-17] Fu J, Zhang Q, Wang P, Zhang L, Tian Y, Li X (2022). Spatio-temporal changes in ecosystem service value and its coordinated development with economy: a case study in Hainan Province, China. Remote Sensing.

[ref-18] Fushita AT, Santos JED, Rocha YT, Zanin EM (2017). Historical land use/cover changes and the hemeroby levels of a bio-cultural landscape: past, present and future. Journal of Geographic Information System.

[ref-19] Geneletti D, Dawa D (2009). Environmental impact assessment of mountain tourism in developing regions: a study in Ladakh, Indian Himalaya. Environmental Impact Assessment Review.

[ref-20] Guo M, Zheng H, Ma S, Zhang M, Zhang M-J, Wang L-J (2024). Developing multiscale landscape planning to mitigate ecological risks: a case study in Nanjing metropolitan area, China. Environmental Impact Assessment Review.

[ref-21] Guo Z, Zhu C, Fan X, Li M, Xu N, Yuan Y, Guan Y, Lyu C, Bai Z (2025). Analysis of ecological network evolution in an ecological restoration area with the MSPA-MCR model: a case study from Ningwu County, China. Ecological Indicators.

[ref-22] Han J, Hu Z, Wang P, Yan Z, Li G, Zhang Y, Zhou T (2022). Spatio-temporal evolution and optimization analysis of ecosystem service value—a case study of coal resource-based city group in Shandong, China. Journal of Cleaner Production.

[ref-23] Hu M, Li Z, Yuan M, Fan C, Xia B (2019). Spatial differentiation of ecological security and differentiated management of ecological conservation in the Pearl River Delta, China. Ecological Indicators.

[ref-24] Jia Y, Tang X, Liu W (2020). Spatial–temporal evolution and correlation analysis of ecosystem service value and landscape ecological risk in Wuhu City. Sustainability.

[ref-25] Jin T, Zhang Y, Zhu Y, Gong J, Yan L (2021). Spatiotemporal variations of ecosystem service values and landscape ecological risk and their correlation in forest area of Loess Plateau, China: a case study of Ziwuling region. Chinese Journal of Applied Ecology.

[ref-26] Kang L, Jia Y, Zhang S (2022). Spatiotemporal distribution and driving forces of ecological service value in the Chinese section of the Silk Road Economic Belt. Ecological Indicators.

[ref-27] Keshtkar H, Voigt W (2016). A spatiotemporal analysis of landscape change using an integrated Markov chain and cellular automata models. Modeling Earth Systems and Environment.

[ref-28] Lan X, Tang H, Liang H (2017). A theoretical framework for researching cultural ecosystem service flows in urban agglomerations. Ecosystem Services.

[ref-29] Li J, Gao M (2019). Spatiotemporal evolution and correlation analysis of ecosystem service values and ecological risk in Binzhou. Acta Ecologica Sinica.

[ref-30] Liu F, Yang L, Wang S (2023). Spatial and temporal evolution and correlation analysis of landscape ecological risks and ecosystem service values in the Jinsha River Basin. Journal of Resources and Ecology.

[ref-31] Lu N, Yang G, Zhang T, Zhang Y, Wu J, Tao L (2023). Spatial and temporal variation of ecosystem service values and correlations of landscapeecological risks in Tokoto County. Journal of Northwest Forestry University.

[ref-32] Luan C, Liu R, Li Y, Zhang Q (2024). Comparison of various models for multi-scenario simulation of land use/land cover to predict ecosystem service value: a case study of Harbin-Changchun Urban Agglomeration, China. Journal of Cleaner Production.

[ref-33] Luo C, Pu S, Yu G (2024). Investigating the impact of climate and land use changes on soil erosion in the Anning River basin in China. Frontiers in Earth Science.

[ref-34] Ma B, Chu Z, Zhou R, Xu B, Wei K, Li B, Zhao T (2023). Longitudinal patterns of fish assemblages in relation to environmental factors in the Anning River, China. Ecological Indicators.

[ref-35] Peng J, Tian L, Liu Y, Zhao M, Hu Y, Wu J (2017). Ecosystem services response to urbanization in metropolitan areas: thresholds identification. Science of the Total Environment.

[ref-36] Peng J, Zong M, Hu Y, Liu Y, Wu J (2015). Assessing landscape ecological risk in a mining city: a case study in Liaoyuan city, China. Sustainability.

[ref-37] Shao H, Liu M, Shao Q, Sun X, Wu J, Xiang Z, Yang W (2014). Research on eco-environmental vulnerability evaluation of the Anning River Basin in the upper reaches of the Yangtze River. Environmental Earth Sciences.

[ref-38] Straton A (2006). A complex systems approach to the value of ecological resources. Ecological Economics.

[ref-39] Tian C, Fang Y, Yang LE, Zhang C (2019). Spatial–temporal analysis of community resilience to multi-hazards in the Anning River basin, Southwest China. International Journal of Disaster Risk Reduction.

[ref-40] Tian J, Gang G (2012). Research on regional ecological security assessment. Energy Procedia.

[ref-41] Wang M, Jiang B, Alatalo JM, Bai Y, Wang Q, Tan J, Ruan J, Su J (2021). Improved ecological monitoring for urban ecosystem protection in China. Ecological Indicators.

[ref-42] Wang L-J, Luo G-Y, Ma S, Wang H-Y, Jiang J, Zhang J-G (2023). Integrating landscape ecological risk into ecosystem service value assessment: a case study of Nanjing City, China. Ecological Indicators.

[ref-43] Wang S, Tan X, Fan F (2022). Landscape ecological risk assessment and impact factor analysis of the Qinghai–Tibetan Plateau. Remote Sensing.

[ref-44] Wang J, Xu C (2017). Geodetector: principle and prospective. Acta Geographica Sinica.

[ref-45] Wang N, Yan J, Su F (2024). Landscape pattern changes and ecological risk assessment of major bays in the Philippines. Ocean & Coastal Management.

[ref-46] Wen S, Cao S, Du M, He Z (2024). Aligning territorial spatial planning with sustainable development goals: a comprehensive analysis of production, living, and ecological spaces in China. Ecological Indicators.

[ref-47] Xia S, Liu Y, Yu X, Fu B (2018). Challenges in coupling LTER with environmental assessments: an insight from potential and reality of the Chinese Ecological Research Network in servicing environment assessments. Science of the Total Environment.

[ref-48] Xie G, Zhang C, Zhang L, Chen W, Li S (2015). Improvement of the evaluation method for ecosystem service value based on per UnitArea. Journal of Natural Resources.

[ref-49] Xie G, Zhang C, Zhen L, Zhang L (2017). Dynamic changes in the value of China’s ecosystem services. Ecosystem Services.

[ref-50] Xing L, Hu M, Wang Y (2020). Integrating ecosystem services value and uncertainty into regional ecological risk assessment: a case study of Hubei Province, Central China. Science of the Total Environment.

[ref-51] Yang J, Xie B, Wang S, Zhang D, Liu C, Mak-Mensah E (2022). Temporal and spatial characteristics of grassland ecosystem service value and its topographic gradient effect in the Yellow River Basin. PLOS ONE.

[ref-52] Yi L, Chen J, Jin Z, Quan Y, Han P, Guan S, Jiang X (2018). Impacts of human activities on coastal ecological environment during the rapid urbanization process in Shenzhen, China. Ocean & Coastal Management.

[ref-53] Zeng X, Huang Y, Xie H, Ma Q, Li J (2024). Impacts of land use and land cover change on the landscape pattern and ecosystem services in the Poyang Lake Basin, China. LandScape Ecology.

[ref-54] Zhang J, Qin G, Cheng S, Wen Y (2023). Spatiotemporal changes and correlation between landscape ecological risk and ecological service value in Hanjiang eco-economic belt. Bulletin of Soil and Water Conservation.

[ref-55] Zhang L, Zhao Y, Yin S, Fang S, Liu X, Pu M (2014). Gradient analysis of dry valley of Minjiang River landscape pattern, based on moving window method. Acta Ecologica Sinica.

[ref-56] Zheng F, Hu Y (2018). Assessing temporal-spatial land use simulation effects with CLUE-S and Markov-CA models in Beijing. Environmental Science and Pollution Research.

[ref-57] Zhou J, Feng B, Wu H, Xu T, Chen L, Zhao X, Guo Q, Li J, Zhang C, Zhu K, Kong Y (2024a). Spatio-temporal evolution and topographic gradient effect of land use and ecosystem service value in the Lhasa River Basin. Journal of Mountain Science.

[ref-58] Zhou J, Feng B, Wu H, Zhang Z, Chen L, Li J, Chen X, Kong Y, Meng Z, Kong X (2025). Spatio-temporal distribution characteristics and driving factors of forest land in the Da-Xiao Liangshan mountains based on topographic gradient. Scientific Reports.

[ref-59] Zhou X, Ji G, Wang F, Ji X, Hou C (2024b). Analysis of the environmental benefits and driving forces of the development of the production–living–ecological space pattern based on the ERI-ESV geodetector. Land.

[ref-60] Zhou J, Wu H, Zhao X, Xu T, Guo Q, Li J (2024c). Topographic gradient differentiation of cultivated land and driving factors of its change in the dry valley of Anning River. Agricultural Research in the Arid Areas.

